# Cytoplasmic Transfer Improves Human Egg Fertilization and Embryo Quality: an Evaluation of Sibling Oocytes in Women with Low Oocyte Quality

**DOI:** 10.1007/s43032-020-00371-8

**Published:** 2020-11-05

**Authors:** Ales Sobek, Emil Tkadlec, Eva Klaskova, Martin Prochazka

**Affiliations:** 1 Fertimed - Infertility Center, 77200 Olomouc, Czech Republic; 2 Olomouc, Czech Republic; 3 Department of Ecology and Environmental Sciences, Faculty of Science, Palacký University Olomouc, 77900 Olomouc, Czech Republic; 4 Department of Paediatrics, University Hospital Olomouc and Faculty of Medicine and Dentistry, Palacký University Olomouc, Olomouc, Czech Republic; 5 Department of Medical Genetics, University Hospital Olomouc and Faculty of Medicine and Dentistry, Palacký University Olomouc, Olomouc, Czech Republic

**Keywords:** Cytoplasmic transfer, Egg quality, Embryo quality, Mitochondria, Mitochondrial donation, Ooplasmic transfer, Ooplasmic transplantation

## Abstract

The aim of this study was to evaluate if cytoplasmic transfer can improve fertilization and embryo quality of women with oocytes of low quality. During ICSI, 10–15% of the cytoplasm from a fresh or frozen young donor oocyte was added to the recipient oocyte. According to the embryo quality, we defined group A as patients in which the best embryo was evident after cytoplasmic transfer and group B as patients in which the best embryo was evident after a simple ICSI. We investigated in the period of 2002–2018, 125 in vitro fertilization cycles involving 1011 fertilized oocytes. Five hundred fifty-seven sibling oocytes were fertilized using ICSI only and 454 oocytes with cytoplasmic transfer. Fertilization rates of oocytes were 67.2% in the cytoplasmic transfer and 53.5% in the ICSI groups (*P* < 0.001). A reduction in fertilization rate was observed with increased women age in the ICSI but not in the cytoplasmic transfer groups. The best embryo quality was found after cytoplasmic transfer in 78 cycles (62.4%) and without cytoplasmic transfer in 40 cycles (32%, *P* < 0.001). No significant differences were detected between the age, hormonal levels, dose of stimulation drugs, number of transferred embryos, pregnancy rate and abortion rate between A and B groups. Cytoplasmic transfer improves fertilization rates and early embryo development in humans with low oocyte quality. All 28 children resulting from cytoplasmic transfer are healthy.

## Background

An increasing number of women over 35 are seeking infertility treatment. There is a higher likelihood of a greater frequency of point mutations in the oocytes in these patients [1]. The egg quality reduces significantly over the age of 35 years or earlier in many circumstances [2, 3]. Mutations in the mitochondrial DNA (mtDNA) occur at a tenfold higher rate than those in nuclear DNA [4]. This is the reason why we expect the first signs of ageing to occur in the cytoplasmic function. Minor mitochondrial dysfunctions [5] can cause a reduction in ATP production or inadequate spindle microtubule production [6] with consequent aneuploidy or maternal age-related trisomy [1].

The number of mitochondria increases from 6000 at the primordial follicle stage to 400,000 or more prior to fertilization (MII oocyte); this increase prepares the oocyte for the increased energy demand during fertilization and early embryo development [3]. Mitochondrial DNA replication stops during fertilization, and each cell division reduces the number of mitochondria in each blastomere by 50%. Consequently, there are only low numbers of mitochondria present in each blastomere at the time when the blastocyst is formed. In contrast, a high number of low-quality mitochondria have been detected in “old” oocytes due to compensatory hyperproduction [5].

The mitochondria guarantee energy production (ATP) for fertilization and early embryonic development [4]. Sperm chromosome cleaning, spindle formation, chromosome fusion and embryo division are all processes that consume large amounts of energy. If the ATP is lacking, then irregularities in chromosome movements can occur, leading to embryo aneuploidy. Cytoplasmic transfer (CT), which involves the transfer of mitochondria, other organelles and a range of ‘unknown cytoplasmic factors’, can increase both the quantity and quality of mitochondria. After the transfer of even a small proportion of young mitochondria, we can expect better direct multiplication of these elements and an improvement in fertilization and early embryonic development [7, 8].

The first animal studies involving CT were carried out in the 1980s [9], and the first human baby born after CT was reported by Cohen [10]. Some other centres have also reported successful treatments with CT [11, 12], although no information is available at present with regard to the effects of CT upon sibling oocytes in a large patient sample. In particular, there is currently no information available regarding improved cytoplasmic function except for appropriate assessments of embryo development [13]. The present study was designed to address this shortfall and to improve our knowledge.

## Methods

### Patients

From 2002 to 2018, 244 patients fulfil the inclusion criteria for the prospective study (age over 35 years, or basal FSH over 12.5 IU/l, or one and more failed IVF/ICSI or less than 5 oocytes retrieved, or high FSH consumption for stimulation over 3000 IU or low ovarian response to stimulation—more than 600 IU FSH/oocyte in current or previous IVF). All patients having stimulation in several stimulation protocols (agonist, antagonist) and have eggs and signed information consent were offered to participate in the study. Patients, giving permission for cytoplasmic transfer only to a smaller part of their eggs were not excluded from the study, because we understand the scepticism about the new method. IVF cycles with all oocytes fertilized using CT (*n* = 119) were not included in this sibling oocyte study. These patients were not further evaluated in this study except referring babies in conclusion.

Out of 125 sibling group participants, 89 patients have experienced 1–7 unsuccessful IVF. In 36 patients without previous IVF failure, 23 were indicated because of age over 35. In the younger age group, 9 had FSH consumption for stimulation over 3000 IU FSH; 5 had less than 5 oocytes retrieved after standard stimulation dose (more than 2500 IU FSH; it means more than 500 IU FSH/oocyte); and 4 suffered high basal FSH (over 12.5 IU/ml). Three young patients suffer from more than 1 indication.

### Cytoplasmic Transfer Method

During ICSI, 10–15% of the cytoplasm from a fresh or frozen young donor oocyte (24–32 years) was added to the recipient oocyte together with the sperm. The cytoplasm from the donor oocyte is taken from the pole far from polar body. First, the sperm is aspirated to the ICSI needle and, later, the cytoplasm from donor oocyte using the same technique and volume, as the aspiration of cytoplasm is done routinely during ICSI. All materials are applied to the recipient oocyte using the same technique as ICSI. The sperm is the mark of the end of donor cytoplasm. One donor oocyte can give a cytoplasm to 4–5 recipient oocytes. All donors were examined for genetic factors (personal interview, chromosomal analysis, cystic fibrosis, thrombophilia), hormonal examination and infectious disease (HIV, hepatitis, lues, chlamydia).

The treatment was allowed by a local ethical committee (1/2001). The only known CT legislation exists in England. The Human Fertilisation and Embryology (Mitochondrial Donation) Regulations, 2015, No. 572 described the indication for “mitochondrial donation” (and associated organelles) in “women who may have mitochondrial abnormalities caused by mtDNA”.

### Fertilization

We aimed to primarily compare the fertilization rate by ICSI with and without CT. The mean patient age was 35.2 years (SE 0.43, range 26–48 years). Out of a total of 1151 oocytes collected from patients, 1011 MII oocytes were fertilized. The randomization of each patient’s oocyte set modifies the non-pair number of mature oocytes, and the right of the patient individually influences the rate of CT/non-CT: 454 oocytes were fertilized with CT (44.9%) and 557 with non-CT (55.1%).

### Embryo Quality

Embryo transfer (ET) was done during the day, when the number of the best embryos is the same as the plan for the transfer. For ET, best-developed embryo(s) from the patient were used, regardless of the method of fertilization. There was no cut-off in the morphologic quality to transfer the embryo. Embryos of all quality were transferred, always using the best of the patient’s embryos (C was better than D; D was transferred in case of no better embryos developed). The blastocyst culture was allowed only in high-quality embryos. Morphologic quality of embryos we can compare together only for embryos on the 2nd and 3rd day of culture, when we distinguish 4 quality levels: A (no dentritus), B (1–10% of dentritus), C (11–30% dentritus) and D (more than 30% dentritus). Days 4 and 5 have another number of quality levels (2 in 96 h or 6 in 120 h of cultivation). Using Gardner’s morphological criteria [14], we determined which embryo from the patient’s set is the best: with CT or without CT. Patients with the best embryo quality after CT were classified as ‘group A’, and patients with the best embryo quality without CT were classified as ‘group B’. Both groups were compared with regard to female age, previous IVF, basal FSH, E2 at the day of HCG ovulation triggering, total number of oocytes recovered, E2 per oocyte, total FSH dose used for stimulation, number of embryos transferred, pregnancy rate, delivery, baby take home rate and number of oocytes (with or without CT) fertilized to have 1 baby.

### Statistical Analysis

Statistically, we first compared fertilization rates between the oocytes with and without CT by applying a test of proportions. Then, we fitted a mixed-effect logistic regression model [15]. To control for the effects of other confounding variables, we included in the model additional predictors: female age, basal FSH, E2 at the day of HCG ovulation triggering, total number of oocytes recovered and total FSH dose used for stimulation.

For the statistical analysis of embryo quality, we applied the simple test of proportion to test whether the proportion of superior embryos with or without CT are equal (*n* = 118). We applied multiple logistic regression models with the probability of a CT embryo being superior to non-CT embryos as a response variable and with the confounding variables (female age, basal FSH, E2 at the day of HCG ovulation triggering, total number of oocytes recovered and total FSH dose used for stimulation) as predictors. Finally, the differences between the groups A and B were tested by a two-sample Wilcoxon test in case of means or by a test of proportions in case of percentages. All statistical analyses were done using R Core [16]. The differences between the means were considered significant at *P* < 0.01.

## Results

**Fig. 1:**
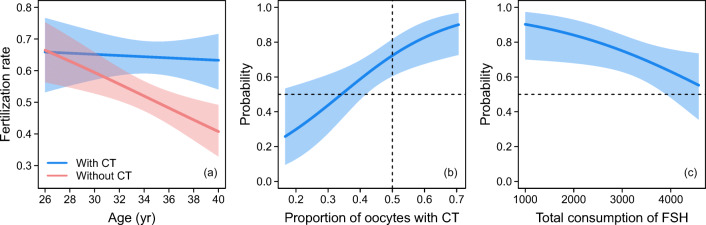
**a** Fertilization rates with and without CT as predicted by the best supported mixed-effect logistic regression model which controlled for the confounding effect of age. The shaded areas indicate the 95% confidence intervals. **b** The probability for CT embryos being superior increased with an increasing proportion of oocytes with CT, as predicted by the best logistic regression model which contained proportions of oocytes with CT and a total dose of FSH used for stimulation. The curve was drawn while keeping a total dose of FSH at the median value. If embryos with and without CT are of equal quality, then the predicted curve should come exactly through the cross of the dashed lines which indicates a probability of 50% for a proportion of 0.5. As the curve intersects the proportion of 0.5 at the probability 0.723, the oocytes subjected to CT clearly result in more embryos of higher quality. **c** The probability for CT embryos of being superior decreases with the increasing total dose of FSH as predicted by the same model while keeping the proportion of CT oocytes at the median value. The probability, however, remains above 0.5 over the whole range of total FSH dose values

There were no significant differences in the fertilization rate in our results after using cytoplasm from fresh (63.4%) or frozen (62.5%) oocytes (*P* = 0.835). Using CT, 434 oocytes (3.6 per woman) were fertilized, and 541 oocytes (4.5 per woman) were fertilized without CT. The fertilization rate in all 125 cycles was 67.2% in the CT group and 53.5% in the non-CT group, with the difference being highly significant (*X*^2^ = 18.9, *P* < 0.001). When applying the logistic regression model to control for the effect of confounding variables, only the age of patients turned out to affect the fertilization rates. This model predicts diverging fertilization rates with patient’s age (Fig. 1a), with the rates decreasing much faster in oocytes without CT. Hence, we conclude that CT treatment improves fertilization rates in oocytes from older patients. As predicted by the model, the rates can even be maintained at a level similar to that in oocytes from younger patients. The effects of other confounding variables, such as female age, basal FSH, E2 at the day of HCG ovulation triggering, total number of oocytes recovered and total FSH dose used for stimulation were not supported by our data.

**Fig. 2:**
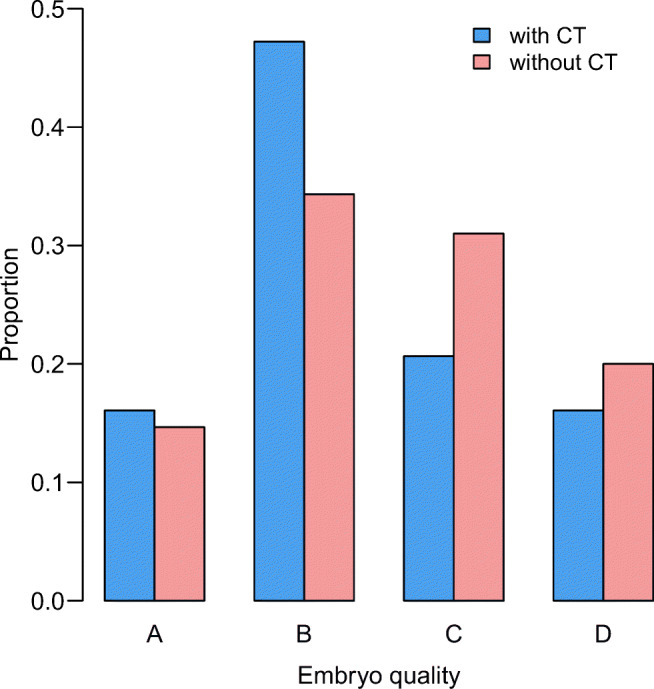
Comparison on embryo quality (day 2 or 3) between groups treated with cytoplasmic transfer (CT) and without cytoplasmic transfer (non-CT). High-quality embryos (A + B) are more often after CT than after non-CT. Statistical significance is in Table 2

Embryos treated with CT have 16% quality A, 47.3% quality B, 20.7% quality C and 16% quality D (Fig. 2). Embryos treated with ICSI only have 14.7% quality A, 34.4% quality B, in 30.6% quality C and 20.1% quality D. High-quality embryos (A + B) are more often after CT (63.3%) than after non-CT (49.3%). The difference is significant (Table 2).

To compare differences between non-CT and CT in one set of sibling oocytes, we identify the best embryo used for embryo transfer in each woman.

We identified seven cycles as being of equal embryo quality after non-CT and CT. These cycles could not be included either in group A or in B because of no differences between best embryo after CT and without CT and were evaluated only about fertilization. The patients in this group were of higher age and had higher basal FSH, higher consumption of stimulation drugs, and lower E2 levels prior to oocyte collection. In five of these cycles (71% and 4% of all cycles), no embryos were produced.

In 78 (66.1%) out of the 118 patients with differences in embryo quality between non-CT and CT treatments, the embryos treated by CT were found to be of superior quality in the patient’s set of embryos (*X*^2^ = 11.1, *P* < 0.001). This result is, however, scaled down because the overall proportion of the oocytes treated with CT was lower than 50%, thereby decreasing the chance for CT embryos to be the best in the patient’s set of embryos. For the equal proportion of CT oocytes of 0.5, the probability for CT embryos being superior is predicted to be 0.723 with the 95% confidence interval (CI) of 0.604 to 0.817 (Fig. 1b). This clearly suggests that the quality of embryos after CT treatment was indeed increased.

We applied multiple logistic models with several predictors, including the proportion of oocytes treated with CT. Two confounding variables turned out to have significant effects on the probabilities: the proportion of oocytes treated with CT and the total dose of FSH. As expected, the probability for CT embryos being superior increased with the increasing proportion of oocytes treated with CT (Fig. 1b), whereas it decreased with the increasing dose of FSH (Fig. 1c). As can be seen from Fig. 1c, the quality of embryos was increased over the whole range of the total FSH dose values, more significantly in IVF cycles with ‘reasonable’ FSH dose used for stimulation (total dose below 3500 IU FSH), but even in cycles with a total FSH dose over 4500 IU, we can detect improvement after CT.

By comparison, the cycles ranked in group A did not differ in any parameters from those in group B (Table 1). We did not find any predictive parameter to specify the group of patients which can benefit from CT or non-CT. From 434 oocytes fertilized by CT, 14 got babies (31 oocytes/baby), and from 541 oocytes fertilized by non-CT only, we got 6 babies (90 oocytes/baby). The difference is significant (*P* = 0.041). The implantation rates of CT only group and non-CT group are not significant (26.7% versus 12.5%) (Table 2).

**Table 1:** Comparison of cycle parameters (SE) between groups A and B showing the best-quality embryos with and without cytoplasmic transfer (CT)

Parameter	A, (*n* = 78) best embryo	B, (*n* = 40) best embryo	Significance	Equal quality
	with CT	without CT	(*P* value)	(*n* = 7)
Mean age of patients (years)	35.3 (0.54)	34.3 (0.79)	0.28	38.1 (1.65)
Age range (years)	26–48	26–46	-	31–45
Previous ART	1.5	1.5	-	1.83
Mean FSH day 3 (IU)	9.20 (0.57)	7.99 (0.55)	0.30	10.71 (2.20)
Mean E2 day of HCG (pmol/l)	6430 (686)	3617 (601)	0.16	4712 (1950)
Mean E2 per 1 oocyte (pmol/l)	705.5 (57.5)	505.9 (54.6)	0.04	903.6 (254.5)
Sum of oocytes for fertilization	664	311	-	36
Mean number of oocytes retrieved	9.32 (0.57)	9.88 (1.03)	0.91	4.75 (0.75)
Mean total dose of FSH (IU)	3175 (112)	3431 (140)	0.21	2859 (438)
Mean FSH dose per oocyte (IU)	466 (39)	535 (64)	0.16	713 (167)
Number of oocytes with CT (%)	314 (43.7)	120 (30.4)	-	20
Number of oocytes without CT (%)	350 (56.3)	191 (69.6)	-	16
Number of embryos transferred	2.2 (0.13)	2.5 (0.10)	0.15	0.9 (0.5)
Number of pregnancies/ET (%)	20 (25.9)	9 (22.5)	0.10	0
Number of miscarriages (%)	7 (35)	3 (33.3)	0.63	-
Number of deliveries (%)	13 (61.9)	6 (66.6)	0.15	-
Baby take home rate (%)	14 (18.2)	7 (17.5)	0.30	-

The data on group of patients with embryos of equal quality are also given

**Table 2:** The number of embryos of different quality is related to the total number of embryos created using the CT and without CT methods

Parameter	With CT	Without CT	(*P* value)	Significance
Total number of embryos	305	300		-
Number of embryos A quality	49 (16%)	44 (14.7%)	*P* = 0.72	NS
Number of embryos B quality	144 (47.3%)	103 (34.4%)	*P* = 0.0017	S
Number of embryos C quality	63 (20.7%)	93 (30.6%)	*P* = 0.0049	S
Number of embryos D quality	49 (16%)	60 (20.1%)	*P* = 0.25	NS
Number of embryos A + B quality	193 (63.3%)	147 (49%)	*P* = 0.0005	S
Number of embryos C + D quality	112 (46.7%)	153 (51%)	*P* = 0.0005	S

Statistical difference was evaluated using Pearson’s chi-squared test. There is a significantly higher proportion of high-quality (A + B) embryos in the CT method and a significantly higher proportion of lower-quality embryos (C + D) in the non-CT method

## Discussion

We did not observe any differences in our results between using the cytoplasm from fresh oocytes between 2002 and 2013 and using the cytoplasm of frozen eggs after 2014. Similar findings were reported by Lanzendorf [11].

We did not observe any differences in terms of age or other parameters between groups A and B. CT can improve egg quality in patients of any age and clinical circumstances (basal FSH, E2, dose of FSH used for stimulation, number of eggs etc.). Figure 1a shows that the fertilization rates with and without CT were equal at an age of around 30 years but decreased subsequently with age without CT; with CT, the fertilization rate remained basically the same as patient age increased. CT eliminated the negative influence of age on egg quality. In this aspect, we need to consider that our treatment group was derived from women with ‘low oocyte quality’, and this is probably the reason that no difference in fertilization occurred between CT and non-CT groups in lower-age patients (below 30 years) than we can expect in standard IVF group (35 years). In 10 patients over 40 years, we detected surprisingly high fertilization rates with CT compared to those without CT; however, we are cautious to interpret this data and excluded it from Fig. 1a because of low number of cases.

The probability for CT embryos to be superior decreases with the increasing total dose of FSH used for stimulation (Fig. 1c). The probability, however, remains above 0.5 over the whole range of total FSH dose values. This explains, in our mind, the role of mtDNA, which is insufficient in our patients with still reasonable ovarian response (lower consumption of FSH used for stimulation), and the role of nuclear DNA which is still good in these patients. In case of high doses of FSH for stimulation (very low ovarian response), a higher amount of nuclear damage can be expected together with mtDNA damage. Better results still exist after CT but not so markedly as in patients with better ovarian function (lower FSH consumption). We inform patients in the consent form that CT improves only cytoplasmic function and cannot repair existing chromosomal abnormalities in either the oocyte or the sperm but can prevent the creation of new problems—incorrect chromosomal fusion. Good results can be expected in cases of good incoming genetic information, which can be linked together with the help of ‘good’ cytoplasm.

Using CT, we obtained a fertilization rate of 67.2%, and after the transfer of embryos with CT, pregnancy rate of 25.9% and baby take home rate of 18.2%. We need to fertilize 31 oocytes to get a baby. The high abortion rate (33–35%) clearly describes our group of patients with low oocyte quality. Darbandi summarized the clinical data arising from six papers and five centres in which the fertilization rate after CT varied between 64 and 85%, and the pregnancy rate varied between 6.4 and 25% [17].

All of the children arising from our work are healthy (14 from sibling oocyte study and 14 from women treated only with CT out of this study). The first is now 15 years old. More information will be given later by our paediatrician. Similar results were published by Chen [18], who reported the good health of 13 children between the ages of 13 and 18 years from Cohen’s study group [19, 20]. Two pregnancies with an abnormal karyotype (45XO) were described [18, 21], but these were related to known age-related dysfunction and not CT-dependent aneuploidy. These authors also referred to one case of autism and one case of learning dysfunction in CT children but concluded that these occurred randomly and were not associated with CT. No such complication occurred in our children. In another study, Malter reported 15 healthy generations after CT in an animal study [22].

Randomization in our study was performed by dividing sibling oocytes into two groups: with and without CT. This process was not 50:50. Patients sometimes asked CT only for a small proportion of their oocytes, and not all number of eggs was even. Despite the disadvantages of the group fertilized by CT, this group was more successful in terms of higher embryo quality. We estimated, using mathematical modelling, that if we had an equal proportion of CT:non-CT cases, then the best embryos would be derived from CT in 72–82% of cycles. Similar information relating to the role of CT in improving embryo quality was published previously [10, 12].

Ovarian function decreases continuously with increasing average life expectancy of women [23]. The age of women seeking ART treatment is increasing worldwide, and there is a clear reduction in oocyte quality over the age of 35 years. Ageing is the first step in causing a reduction in cytoplasmic function, predominantly mitochondrial activity [24, 25]. Our centre began to offer CT in 2002 and offered patients the option of transferring ‘unknown cytoplasmic factors’, cellular organelles and enzymes to patient’s oocyte. Darbandi [17] described similar factors, which they referred to as ‘undetected factors’. In recent years, many publications have reported the role of mitochondria in oocyte quality, fertilization and early embryo development [26–30].

In humans, mutant and normal (wild-type) mtDNA co-exist. If the proportion of mutant mtDNA is less than 10–18%, then no clinical symptoms are present. However, between 18 and 80%, we can expect a reduction in cytoplasmic function. First, the damage is detectable during high energy-dependent actions (such as fertilization and cell division). If the number of mutations in one mtDNA region exceeds 70–80%, then mitochondrial disease can be detected, and clinical symptoms can be diagnosed [5]. Oocytes with a low number of mtDNA are not able to ovulate spontaneously, although IVF hyperstimulation gives them a chance of maturing [4]. In this way, we can produce many low-quality oocytes in older women and women suffering from premature ovarian failure. CT can correct unspecified ooplasmic deficiency.

Heteroplasmy has been reported as a normal occurrence in human oocytes, early embryos and the human body [31, 32]. Data also showed that the artificial induction of heteroplasmy after CT treatment did not result in any health problems for the children [21]. All eggs containing a proportion of mutated mtDNA and all embryos containing surviving sperm mtDNA are heteroplasmic. Because mtDNA does not follow the normal Mendelian pattern of inheritance and is only inherited from the mother [4], there is a need for changes to guarantee development from one generation to another comparable with nuclear DNA (father and mother fusion). These changes ensure in mtDNA spontaneous mutations and their irregular distribution by a ‘bottleneck effect’ [28, 32]. This bottleneck distributes ‘good’ and ‘bad’ quality mtDNA to daughter cells in an irregular fashion, thus providing many different combinations, which can be classified generally as only good, mostly good or mostly wrong. Poor-quality cells are unable to develop properly and can be eliminated by cellular death or can be extruded out of the embryo [25]. In this way, mutated mtDNA can be eliminated from a family in one [4] to four populations [32]. The irregular distribution of mtDNA through the bottleneck effect can cause differential distribution of mtDNA mutations in different cell populations, a problem known as chimerism [26]. This can create discrepancies in the evaluation of mtDNA in different tissues [33]. Following a rapid increase in our knowledge in the field of mitochondrial mutations, we now have a better understanding of how CT can improve cytoplasmic activity during fertilization. Heteroplasmy in human cells after CT (less than 50%) has only been partly found in human offspring [18, 27], differs in babies of different ages [26, 27], and can decrease with age [33] or disappear completely [10]. Heteroplasmy of mtDNA has been used as a means of identifying Romanov family members, the ratio of heteroplasmy being different in each family member between Tsar Nicholas II and his brothers [31].

CT is a new technology, which can increase the number and quality of mitochondria in oocytes during fertilization. Recently, we have also begun to discuss the role of communication between the mitochondria and the nucleus [25, 27], mitochondrial transport between cells [20, 26], mitochondrial extrusion to the extracellular space [20], Golgi elements and the endoplasmic reticulum, Baldini bodies [32], epigenetic effects [17], melatonin synthesis in mitochondria [25], Ca^2+^ oscillations [4, 13], enzymatic repair processes [17], mitochondrial autophagy/mitophagy [34] and many other processes. Such information can provide an explanation as to why some procedures (e.g. CT) are beneficial.

Now, we can also exclude the possibility of transferring mitochondrial disease with a small amount of cytoplasm. The donor of the cytoplasm is healthy. The donor cytoplasm is not expected to contain more than 5–10% of mutations, which cannot, after dilution in the recipient cytoplasm, create any problems. The probability that mutations in donor mtDNA are the same as mutations in recipient mtDNA is lower than the chances of two rare recessive disease carriers becoming parents together. CT can thus prevent mitochondrial disease and chaotic DNA mosaicism [17, 20]. Transferred mtDNA involves only a very low number of genes [9], which do not influence the phenotype of the offspring and are not detected in many of these babies.

CT is indicated in women with reduced egg cell quality. Its advantage for a woman is to increase the likelihood of getting her own baby over treatment without CT. However, this probability certainly does not reach the probability of conceiving by donated oocytes, where a woman loses her genetic continuity.

We agree with Edwards: in the context of the likelihood that a fertilized egg will develop to birth, it often comes as a surprise to those not involved in the study of early human development that reproduction in our species is an inherently inefficient process [35]. We hope that our experiences can allow a greater understanding of this complex procedure.

## Conclusions

Using sibling oocytes, we demonstrated that cytoplasmic transfer improves fertilization rates and early embryo development in women with low ovarian function. All 28 children resulted after CT are healthy. There is an urgent need to offer patients a procedure to improve egg quality in cases not only involving a higher maternal age but also where other infertility treatments have failed. CT is the method of choice in cases of poor oocyte quality, which in many cases may delay the need for indication of egg donation.

## Data Availability

The dataset analysed during current study are available in the Tkadlec and Sobek repository after request.

## Abbreviations

ATP: adenosine triphosphate
CT: cytoplasmic transfer
DNA: deoxyribonucleic acid
ET: embryo transfer
E2: estradiol
FSH: follicle-stimulating hormone
HCG: human chorionic gonadotropin
ICSI: intracytoplasmic sperm injection
IVF: in vitro fertilization
MII: oocyte in metaphasis II
mtDNA: mitochondrial DNA
